# A fast and robust hippocampal subfields segmentation: HSF revealing lifespan volumetric dynamics

**DOI:** 10.3389/fninf.2023.1130845

**Published:** 2023-06-15

**Authors:** Clement Poiret, Antoine Bouyeure, Sandesh Patil, Antoine Grigis, Edouard Duchesnay, Matthieu Faillot, Michel Bottlaender, Frederic Lemaitre, Marion Noulhiane

**Affiliations:** ^1^UNIACT, NeuroSpin, CEA Paris-Saclay, Frederic Joliot Institute, Gif-sur-Yvette, France; ^2^NeuroSpin, CEA Paris-Saclay, Frederic Joliot Institute, Gif-sur-Yvette, France; ^3^InDEV, NeuroDiderot, Université Paris Cité, Inserm, Paris, France; ^4^BioMaps, Service Hospitalier Frédéric Joliot, CNRS, Inserm, Université Paris-Saclay, Orsay, France; ^5^CETAPS EA 3832, Université de Rouen, Rouen, France; ^6^CRIOBE, UAR 3278, CNRS-EPHE-UPVD, Mooréa, France

**Keywords:** deep learning, semantic segmentation, MRI, development, aging

## Abstract

The hippocampal subfields, pivotal to episodic memory, are distinct both in terms of cyto- and myeloarchitectony. Studying the structure of hippocampal subfields *in vivo* is crucial to understand volumetric trajectories across the lifespan, from the emergence of episodic memory during early childhood to memory impairments found in older adults. However, segmenting hippocampal subfields on conventional MRI sequences is challenging because of their small size. Furthermore, there is to date no unified segmentation protocol for the hippocampal subfields, which limits comparisons between studies. Therefore, we introduced a novel segmentation tool called HSF short for hippocampal segmentation factory, which leverages an end-to-end deep learning pipeline. First, we validated HSF against currently used tools (ASHS, HIPS, and HippUnfold). Then, we used HSF on 3,750 subjects from the HCP development, young adults, and aging datasets to study the effect of age and sex on hippocampal subfields volumes. Firstly, we showed HSF to be closer to manual segmentation than other currently used tools (*p* < 0.001), regarding the Dice Coefficient, Hausdorff Distance, and Volumetric Similarity. Then, we showed differential maturation and aging across subfields, with the dentate gyrus being the most affected by age. We also found faster growth and decay in men than in women for most hippocampal subfields. Thus, while we introduced a new, fast and robust end-to-end segmentation tool, our neuroanatomical results concerning the lifespan trajectories of the hippocampal subfields reconcile previous conflicting results.

## 1. Introduction

Episodic memory, the memory of specific episodes with spatiotemporal details, is critically underpinned by the hippocampal subfields, namely the dentate gyrus (DG), cornu ammonis from 1 to 3 (CA1/2/3), and the subiculum. Each subfield presents a distinct myelo- and cyto-architectony, and plays a critical role in episodic memory functions. For example, the DG and CA3 are involved in pattern separation, which allows the storage and retrieval of similar but distinct events (Yassa and Stark, 2011). CA1 and subiculum are necessary for pattern completion, i.e., the reconstruction of a full memory from partial elements. Since episodic memory performance correlates with variations in hippocampal subfields volume (Palombo et al., 2018), we hypothesize that hippocampal subfields volumetric trajectories are associated with the evolution of episodic memory performance across the lifespan.

Analyzing hippocampal subfields' dynamics implies delineating their boundaries, boundaries often defined at a microscopic scale. Unfortunately, Magnetic Resonance Imaging (MRI) cannot study the unique myelo- and cyto-architectures of subfields, because structures such as CA1 and the Subiculum have the same contrast (Yushkevich et al., 2015a). Numerous efforts have been made to use geometrical heuristics to map histological features to MRI, thereby providing manual segmentation guidelines (Berron et al., 2017; Dalton et al., 2017). Manual segmentation with these protocols is now considered the gold standard for studying the hippocampal subfields *in vivo*. However, it is a complex, time-consuming, and subjective task which makes it error-prone and limits reproducibility. MRI segmentation of hippocampal subfields faces multiple difficulties, mainly caused by a lack of resolution, tissue ambiguity (notably in the head and the tail of the hippocampus), and noise. This problem is amplified by the lack of standardized segmentation protocols. For example, some protocols merge CA1, 2, and 3, sometimes delineating a separate CA4 or even excluding the hippocampal head or tail. This leads to multiple divergent protocols, inducing a lot of variabilities, notably in the boundary between DG and CA3, 4, and the boundary between CA1 and the subiculum with inter-protocol differences of almost 2 mm (Yushkevich et al., 2015a).

Recent efforts have been made to uniformize and automatize the hippocampal subfields segmentation task (Yushkevich et al., 2015a; Wisse et al., 2017). New hippocampal subfields' segmentation tools have recently been developed, such as ASHS (Yushkevich et al., 2015b), HIPS (Romero et al., 2017), or even more recently HippUnfold (DeKraker et al., 2021). They provide better segmentations, closer to manual segmentation, but neither of them implements state-of-the-art end-to-end deep learning which has been proven to be more fault-tolerant and adaptable to new observations, especially on complex and non-linear tasks (O'Mahony et al., 2020). Recent studies highlighted the possible gains of end-to-end deep learning for hippocampal segmentation (Qiu et al., 2019; Zhu et al., 2019; Yang et al., 2020), promising fast inference time (less than a minute per subject against several hours for FreeSurfer), higher accuracy, and higher robustness to anatomical variations. Unfortunately, most deep learning solutions are currently provided as a proof-of-concept, with either no public implementation, no pre-trained models, or are trained on small and specific datasets limiting generalizability. The current literature lacks an end-to-end deep learning segmentation protocol trained on a heterogeneous database to ensure segmentation quality across (i) contrast, (ii) magnetic field intensity, (iii) age range, or (iv) health condition.

Even though segmentation protocols still need to be uniformized, there is a disparity of available segmentation tools for the hippocampal subfields. The current understanding of the effect of age and sex on volumetric changes in hippocampal subfields across the lifespan is based on manual or (semi-)automatic segmentation studies. Uematsu et al. (2012) found that the total hippocampal volume is increasing until early adulthood. Another study showed a differential maturation between the posterior and anterior hippocampal portions (Gogtay et al., 2006). Regarding sex difference, Suzuki (2004) showed that the myelination process, which is thought to contribute to the increase in volume during adolescence, takes place earlier in women (i.e., before the age of 18) than in men (i.e., after the age of 20), with a potentially more pronounced developmental dynamic in men than in women. Ziegler et al. (2012) noted an increase of gray matter volume during adulthood in the hippocampus up to 41 years old, with a maximum at 62 years old for the DG and CA, followed by fast atrophy. This is in accordance with Yang et al. (2013), who identified a quadratic relationship between the overall volume of the hippocampus and age, with an inflection point at 63 years old, followed by a strong negative correlation between volume and age.

Non-human primate studies (e.g., 20) have shown that subfields such as the DG, CA2/3, and the subiculum (but not the pre- nor para-subiculum) are growing asynchronously until adulthood. However, this question has only been recently addressed in human children and adolescents with inconsistent results. According to Ellis et al. (2021), the DG exhibits a very rapid growth in infants, doubling in size, associated with an increase in CA1 and CA3 volumes during development (8–14 years old). This contrasts with a stable or a slight linear decrease in subicular volumes (Ziegler et al., 2012; Lee et al., 2014). Concerning normal aging, data suggest a volumetric decrease of all subfields which predominates in the DG (de Flores et al., 2015; Foster et al., 2019). While the literature suggests a differential maturation and aging of hippocampal subfields, there is currently a lack of accurate automated segmentation tools which hinders the use of large datasets to study trajectories across the lifespan.

Here, we offer the first end-to-end deep learning pipeline to segment the hippocampal subfields. Hippocampal segmentation factory (HSF) is an open-source tool that leverages new computer vision segmentation methods. It was trained on a heterogeneous database comprising all public datasets with manually segmented hippocampal subfields and new manually segmented observations to ensure generalization. We hypothesized (i) that HSF provides a better overlap (dice coefficient), fewer outliers (Hausdorff distance), and a better volumetric similarity than currently available tools abiding Barron's protocol (Berron et al., 2017); (ii) that subfields such as DG and CA1 exhibit differential lifespan dynamics which can be divided into three periods (a. growth, b. stability, and c. decay); (iii) a fast decay for all subfields starting from 60 to 65 years old; (iv) finally that there are sex differences in the volumetric trajectories, with volumetric variations being more intense in men than in women.

## 2. Method

This section aims at describing (i) the technical details of HSF development in terms of computational architecture, training regime, and inference peculiarities, (ii) how it differs from other state-of-the-art tools addressing the same segmentation problem, and (iii) how we leveraged the potential of HSF to study hippocampal subfields volumetric trajectories in large healthy individuals datasets which covers the lifespan (5–100+ years old). Please note that we conducted a speed test comparing the tools included in our benchmark. While FreeSurfer, one of the most used neuroimaging tools, possesses modules for hippocampal subfields segmentation, we chose not to compare it. Although FreeSurfer (Iglesias et al., 2015) is still considered a classic neuroimaging tool, it has recently incorporated deep learning-based approaches. Because it has useful automated features outside the scope of this study, it produces many outputs leading to a long computing time, which makes it inconvenient for scientific studies interested in a single substructure of the human brain: its inference time of approximately 10 h per subject is slower than manual segmentation of the hippocampal subfields. While previous studies found the segmentation quality of FreeSurfer to be good enough to study the hippocampal subfields (Schmidt et al., 2018), others have demonstrated that FreeSurfer has poorer segmentation quality in comparison to the tools included in our benchmark (de Flores et al., 2015; DeKraker et al., 2022), with segmentations that are in a mismatch with known anatomical boundaries leading to a significantly different volumetry, especially in the head and the tail of the hippocampus (Wisse et al., 2014). Thus, as we are only interested in fast tools only tackling hippocampal subfields segmentation, we only used FreeSurfer as a benchmark for speed comparison.

### 2.1. HSF: description of the hippocampal segmentation factory

HSF is designed to be a fully customizable end-to-end pipeline, handling tasks from the preprocessing of raw anatomical images, to the segmentation of the hippocampal subfields through specialized and highly efficient deep learning models comprised in a “Model Hub” on any hardware acceleration platform such as CUDA, TensorRT, or OpenVINO. HSF also supports the DeepSparse compute engine to benefit from the AVX512 (VNNI) vector instruction set. HSF is distributed under the MIT license at https://github.com/clementpoiret/HSF.

#### 2.1.1. Datasets description

The key strength of HSF lies in its training database, which consists of 12 datasets of manually segmented hippocampi by individual expert raters (Table 1), totaling 411 subjects.

**Table 1:** Training datasets used for HSF.

**Dataset**	**Contrasts**	**Subfields**	**Field**	**Age**	**Condition**
Winterburn et al. (2013)	T1 and T2	DG/CA/Sub	3T	29–57	-
Kulaga-Yoskovitz et al. (2015)	T1 and T2	DG/CA/Sub	3T	21–53	-
Yushkevich et al. (2015b)	T1 and T2	DG/CA1/CA2-3/Sub	3T		MCI
Hindy et al. (2016)	T2	DG/CA1/CA2/CA3/Sub	3T	18–30	-
Bouyeure et al. (2021)	T1 and T2	DG/CA1/CA2-3/Sub	3T	4–12	-
Yushkevich et al. (2010)	T1 and T2	DG/CA1/CA2/CA3/Sub	4T	38–82	MCI/AD
HIPlay7^*^	T1 and T2	DG/CA1/CA2/CA3/Sub	7T	12–21	TLE
Wisse et al. (2016)	T2	DG/CA1/CA2/CA3/Sub	7T	50–68	-
Berron et al. (2017)	T1 and T2	DG/CA1/CA2/CA3/Sub	7T	19–32	-
Haeger et al. (2020)^**^	T2	DG/CA1/CA2/CA3/Sub	7T	50–70	-
Shaw et al. (2020)^**^	T1 and T2	DG/CA1/CA2/CA3/Sub	7T	23–29	-
Lagarde et al. (2021)^**^	T2	DG/CA1/CA2/CA3/Sub	7T	50–84	SC/MCI/AD

Description of the training database, alongside their manually segmented hippocampal subfields, namely the dentate gyrus (DG), the cornu ammonis (CA)1, CA2, and CA3. Included participants are either healthy, exhibiting mild cognitive impairments (MCI), Alzheimer's disease (AD), hippocampal sclerosis (SC), or temporal lobe epilepsy (TLE).

* In-house dataset, ANR-16-NEUC-0001-01; Manual Segmentation on 23 controls and 4 temporal lobe epilepsies; 1 mm T1w and 0.125^*^0.125^*^1.2 mm T2w MRIs.

** Manual segmentations on 7 subjects per dataset performed by the authors (CP, SP, MF, MB, and MN), following Berron et al. (2017).

#### 2.1.2. Internal information processing

**Figure 1:**
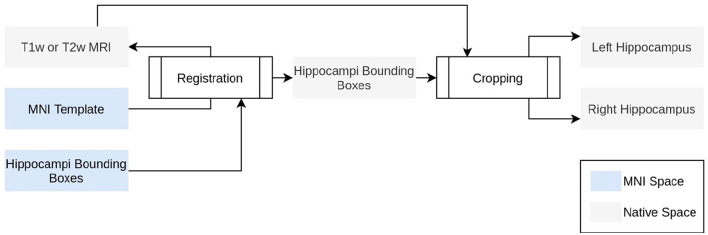
Technical description of ROILoc. ROILoc aims at locating and extracting any region of interest on a given MRI.

**Figure 2:**
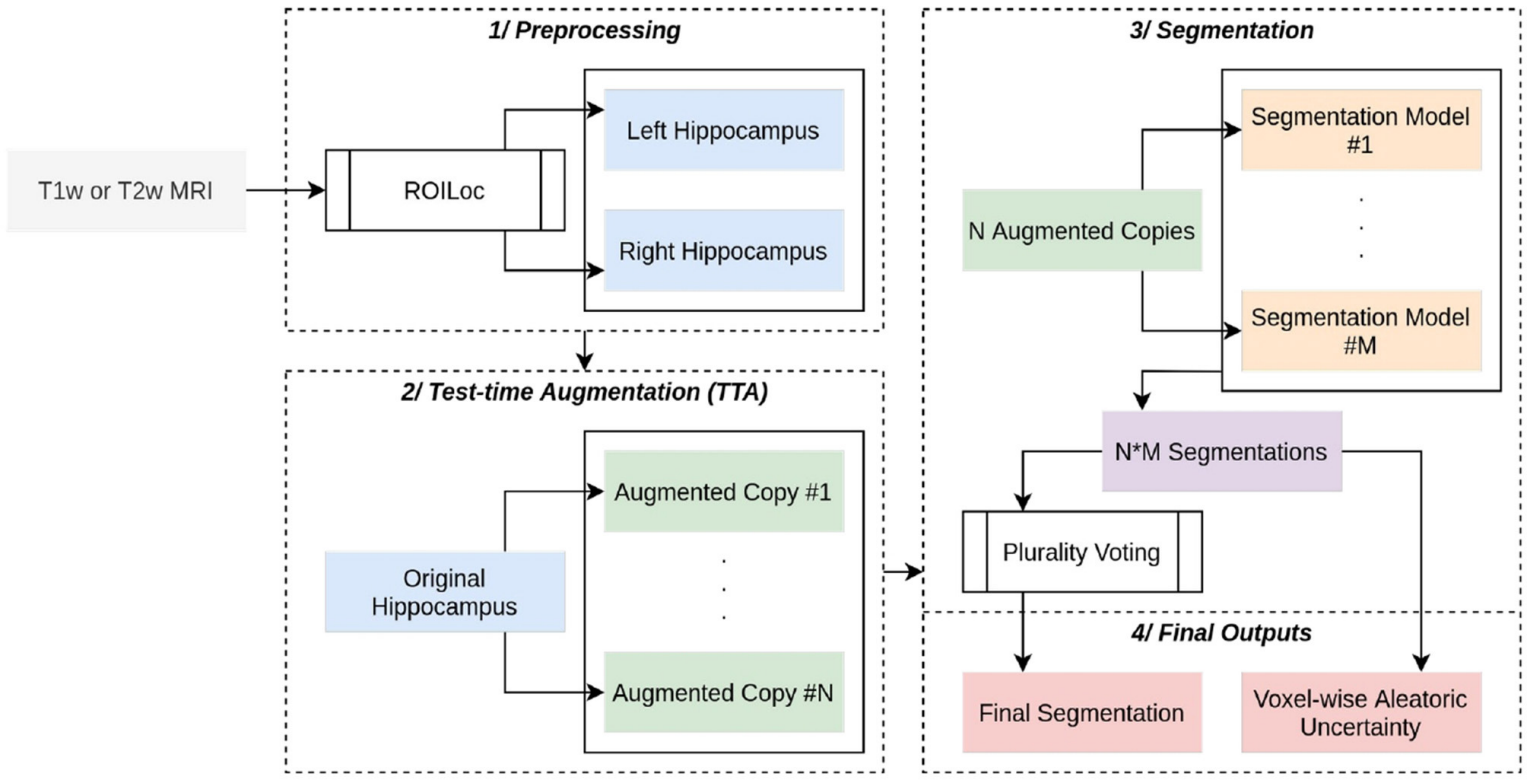
Complete overview of HSF. A (T1w or T1w) MRI passes through ROILoc to extract the left and right hippocampi. Each subvolume is then randomly augmented to obtain 21 different versions of the same hippocampus. Each segmentation goes through five independent deep learning models, and the final segmentation is a voxel-wise plurality vote across all segmentations. A voxel-wise aleatoric uncertainty map is computed for further post hoc analysis.

The HSF pipeline consists of three main steps: 1/a preprocessing step handled by ROILoc (a standalone by-product of HSF available at https://github.com/clementpoiret/ROILoc) to extract the hippocampi from a given MRI (Figure 1), 2/an augmentation pipeline, and 3/a segmentation by multiple expert models in order to produce both the segmentation and an uncertainty map (Figure 2).

In order to limit the computational impact of HSF, we used a preprocessing step to extract the hippocampi from the MRI. To do so, ROILoc registers the MNI152 09c Sym template (Fonov et al., 2009) to the T1w or T2w input MRI. Utilizing the CerebrA atlas (Manera et al., 2020), the registration process facilitated the inference of approximate coordinates of the hippocampus in native space. ROILoc then crops the MRI into two volumes corresponding to the right and left hippocampi from head to tail, with an arbitrary safety margin. To finish the preprocessing, the resulting crops are Z-normalized and padded to obtain shapes that are multiple of 8 to satisfy hardware acceleration constraints.

HSF provides a “Model Hub” offering multiple pre-trained models that can handle preprocessed hippocampi. Our built-in models are 3D Residual UNets of depth 4, with ResNet building blocks (Zhang et al., 2018) and transposed convolutions as the upsampling method. We have replaced the additive skip connections with a self-attention mechanism inspired by the one introduced for 2D images by Oktay et al. (2018), with BatchNorm layers replaced by SwitchNorm layers (Luo et al., 2019). Each segmentation model has its efficient counterpart that can benefit from the AVX512-VNNI instruction set due to pruning (at 70%) and int8-Quantization through NeuralMagic's SparseML.

#### 2.1.3. Training methodology

To augment the quality of the segmentation, we employed the widely used technique called bagging. We trained five “weak-learner” models, each of which was generated by random sampling, with replacement, N samples from the original training set, which contained 822 hippocampi. The bagging technique then amalgamated each weak learner into a strong learner, which displayed a superior accuracy of prediction compared to each weak learner on its own. Bagging outperforms the conventional random split because it introduces more variability (i.e., some subjects can be observed multiple times during a single epoch), thereby enhancing the prediction of the strong learner (Opitz and Maclin, 1999). Each model is trained with an AdamW optimizer, a one-cycle learning rate scheduler, and stochastic weight averaging for 512 epochs with a batch size of 1 to handle heterogeneous input volumes. int8-Quantized models are trained with quantization-aware training.

Given an input *x*, our segmentation loss *L* is defined with *TP* and *TN* the true positives and negatives, *FP* and *FN* the false positives and negatives, and α = 0.3, β = 0.7, $\gamma =\frac{3}{4}$ such as:


$$\begin{array}{l} L={\left(1-\frac{TP}{TP+\beta FN+\alpha FP}\right)}^{\gamma } \end{array}$$

While the base loss function is a focal Tversky, the loss function was modulated for each observation to handle different segmentation protocols. As HSF predicts CA1, CA2, and CA3, we merged classes (e.g., CA2 and CA3) at training time to learn from observations that do not distinguish them. For segmentation protocols having a separate head or tail class, all predictions are merged to form a single ‘hippocampus' class so that predicting any subfield outside the ‘head' or ‘tail' class is penalized but not inside of them.

#### 2.1.4. Inference

To further enhance the segmentation pipeline, test-time augmentation is natively implemented, augmenting each hippocampus with random horizontal flips, and with affine and elastic deformations. The final segmentation is computed as a voxel-wise plurality vote, assigning to a given voxel the most frequent class. For the sake of further *post hoc* analysis of the segmentation quality, a voxel-wise aleatoric uncertainty *H*(*Y*^*i*^∨*X*) is also computed (Wang et al., 2019). Given a set *Y* of *i* predictions, in HSF:


(1) $$\begin{array}{l} H\left(Y^{i}\vee X\right)\approx -\overset{M}{\underset{m=1}{\sum }}\hat{p_{m}^{i}}\ln\hat{p_{m}^{i}}\; \end{array}$$

where $\hat{p_{m}^{i}}$ is the frequency of the *m*^*th*^ unique value in *Y*^*i*^.

### 2.2. Benchmarking HSF against ASHS, HIPS, and HippUnfold

HSF has been assessed against the most recent and widespread tools for hippocampal segmentation: ASHS (Yushkevich et al., 2015b), HIPS (Romero et al., 2017), and HippUnfold (DeKraker et al., 2021). To compare it with manual segmentations, CP, AB, SP, and MF randomly segmented 25 subjects who were excluded from our training set from 5 different datasets: HiPlay7, MemoDev (Bouyeure et al., 2021) (Table 1), as well as HCP-Development (HCP-D), HCP-Young Adults (HCP-YA), and HCP-Aging (HCP-A). This segmentation process took approximately 5 h per hippocampus. In relation to an earlier study on MemoDev, an assessment was conducted by Bouyeure et al. (2021) to determine the reliability of the manual segmentations. This evaluation involved the computation of an inter-rater reliability index, specifically the dice coefficient, between two individual tracers, who followed the same segmentation protocol. Furthermore, it is worth noting that both raters had no prior knowledge of the participants' age, sex, or memory performance. The obtained inter-rater reliability indices were notably high at 0.77 and 0.79 for the right and left hippocampi, respectively. Segmentations are compared on three metrics:

- the dice coefficient (DC), an overlap metric ranging from 0 (no overlap) to 1 (full overlap) defined as $DC=\frac{2\left|y_{m}\cap y_{p}\right|}{\left|y_{m}\right|+\left|y_{p}\;\right|}$,
- the Hausdorff distance (HD), a metric of surface distance ranging from 0 to +inf. With the directed Hausdorff distance between two point sets *X* and *Y* such as $hd\left(X,Y\right)=\begin{array}{c} \max\\ x\in X \end{array}\begin{array}{c} \min\\ y\in Y \end{array}||x-y{||}_{2}$, the HD is defined as *HD*(*y*_*m*_, *y*_*p*_) = *max*(*hd*(*y*_*m*_, *y*_*p*_), *hd*(*y*_*p*_, *y*_*m*_) ),
- and the volumetric similarity (VS), a comparison between volumes of two segmentations ranging from 0 (complete dissimilarity between volumes) to 1 (exact match between volumes). With the volume of a region *S*, it is defined as $VS=2\frac{\left|S_{m}\cap S_{p}\right|}{\left|S_{m}+S_{p}\;\right|}.100\%$.

As both T1w and T2w images can be segmented by HSF, we conducted an additional analysis to evaluate any discrepancies in quality across these contrasts using the same metrics. Given the strong correlation between contrast and resolution (e.g., an isometric millimetric MPRAGE 3D T1w and anisotropic 2D Coro-T2w), we limited our study to only 15 subjects from our test set sourced from the HCP databases, where T1w and T2w MRIs are in the same space and at the same resolution. Owing to the presence of either heteroscedasticity or non-normal distributions of scores, we compared segmentations utilizing non-parametric Kruskal–Wallis or pairwise Wilcoxon–Mann–Whitney tests, with p-values corrected using the Benjamini–Hochberg false discovery rate.

### 2.3. HSF: analyzing the Human Connectome Project

The following sections are specifically dedicated to explaining how we used HSF (process and inference) to study hippocampal subfields trajectories across the lifespan in the HCP datasets (HCP-D, HCP-YA, and HCP-A).

#### 2.3.1. Datasets descriptions

All databases are acquired on a 3T Siemens Prisma (Skyra for HCP-YA) scanner:

- **HCP-D**: HCP-D contains 1350 healthy children, adolescents, and young adults aged from 5 to 21 years. T1w and T2w MRIs are acquired at an isotropic resolution of 0.8 mm across four sites (Somerville et al., 2018),
- **HCP-YA**: HCP-YA includes 1,200 subjects with ages ranging from 22 to 35 years. T1w and T2w MRIs have been acquired on a single site at an isotropic resolution of 0.7 mm,
- **HCP-A**: HCP-A comprises 1,200 subjects from 36 to 100+ years old. T1w and T2w MRIs are acquired at an isotropic resolution of 0.8 mm across four different sites (Bookheimer et al., 2019).

The HCP datasets were provided in part by the Human Connectome Project, WU-Minn Consortium (Principal Investigators: David Van Essen and Kamil Ugurbil; 1U54MH091657) funded by the 16 NIH Institutes and Centers that support the NIH Blueprint for Neuroscience Research and by the McDonnell Center for Systems Neuroscience at Washington University.

#### 2.3.2. MRI segmentation

Prior to HCP's datasets' segmentation and after the HSF validation, we retrained HSF's models with the manually segmented observations coming from the previous section (see Section *2.2*.) including observations from the HCP's datasets. We thereby improved the reliability of segmentations by including new and HCP-specific observations to ensure there was no mismatch between our training set's distribution and HCP's distribution of observations. All segmentations are performed on T2w images, with ROILoc's location algorithm using the ‘Affine‘ registration and a margin of 16 voxels in all directions to ensure that whole hippocampi are included in their boxes.

#### 2.3.3. Lifespan modeling

The whole hippocampus and each subfield were modeled for each sex as a natural cubic spline (NCS) regression between age and volume, a flexible, simple, and efficient model to describe trends (Greenland, 1995; Elhakeem et al., 2022). Cubic models have been validated to study developmental trajectories of the amygdala and the whole hippocampus (Uematsu et al., 2012; Bussy et al., 2021). NCS allowed us to model the growth and decay of hippocampal subfields by fitting a set of piecewise polynomial regressions smoothly joining at points called knots, with a linearity constraint at the extremity of the curve. Significance and goodness of fit for the NCS are computed similarly to linear regressions because NCS are fitted using an ordinary least-squares algorithm. We chose the number of degrees of freedom by minimizing an Akaike Information Criterion. Then, inflection points in the volumetric trajectories of the ROIs were detected as suggested by Satopaa et al. (2011). Finally, we computed an anteroposterior evolution of the subfield's volume on a per-slice basis averaged across every subject.

#### 2.3.4. Statistical analysis

Although lifespan dynamics of the hippocampus and its subfields are thought to be non-linear (e.g., 15,18,34), we assume that within a single period, defined as the uninterrupted period between two distinct inflection points (e.g., young adults), the relationship between age and volume is linear. Therefore, for each lifespan period, we tested (i) the relationship between age and volume, (ii) the relationship between sexes, and (iii) the interaction between these two independent variables using an ordinary least-squares regression. *P*-values are corrected using a Benjamini–Hochberg false discovery rate.

## 3. Results

### 3.1. Benchmarking HSF against ASHS, HIPS, and HippUnfold

**Figure 3:**
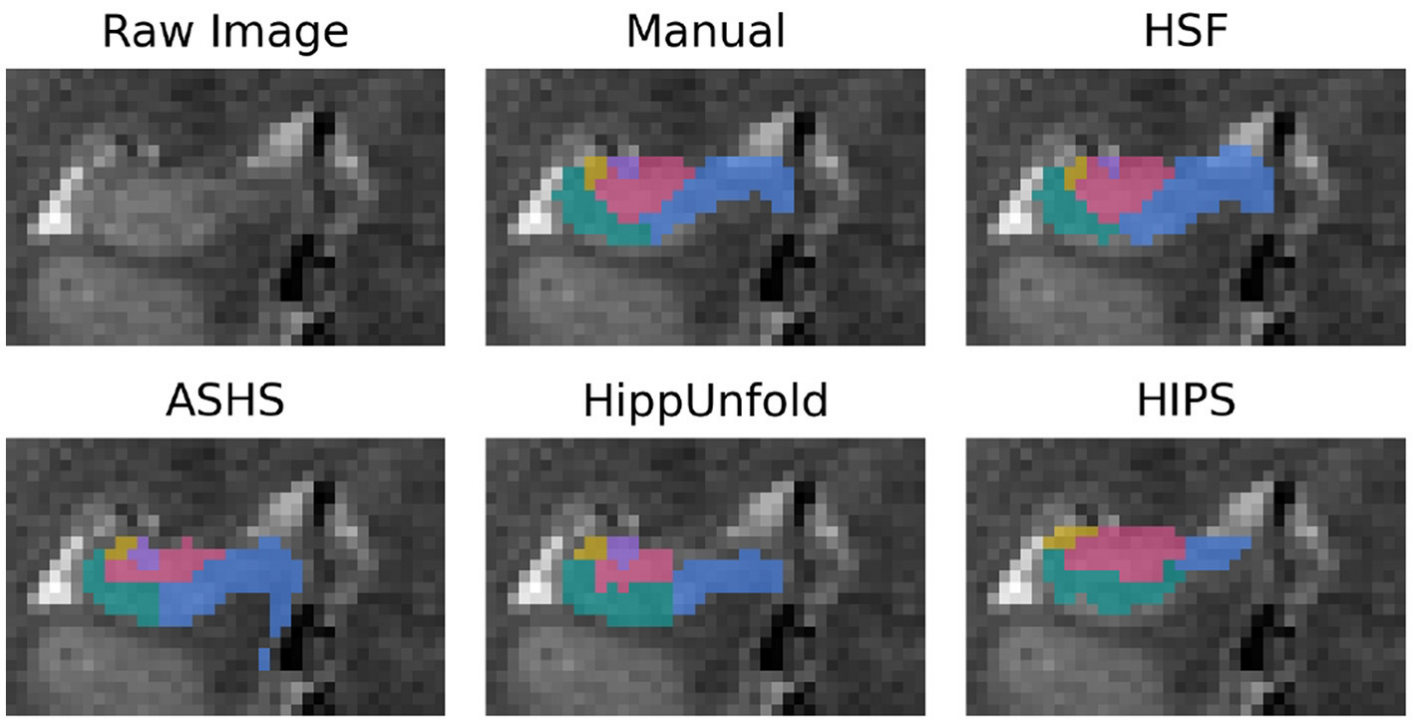
Segmentation example from a random subject. The dentate gyrus is in red, CA1/2/3 are in green, yellow, and purple, and the subiculum is in blue.

First, we validated HSF against three state-of-the-art hippocampal subfields segmentation tools: ASHS, HIPS, and HippUnfold (Figure 3). While manual segmentation may require up to 5 h per subject, FreeSurfer 7 may take even longer, exceeding 10 h due to its all-inclusive pipeline, encompassing whole-brain segmentation and cortical morphometry. As we were interested solely in hippocampal subfields segmentation, we have compared only the specialized tools, which were, therefore, much faster: HIPS, ASHS, and HippUnfold can segment a new subject in under an hour. HSF is even faster, taking only minutes to segment a new subject from the HCP. While HIPS requires the use of the volBrain service and can take up to a day to complete due to queueing, HSF is much quicker. In its most accurate mode, HSF takes only 5 mi on a CPU and 90 s on an NVIDIA A100 GPU (Table 2). In its fast mode, HSF can segment a new subject in only 15 s on both CPU and GPU, with the main speed bottleneck being the registration tool ANTs, which is used to localize the hippocampus (ROILoc).

**Table 2:** Segmentation time of reference software vs. manual segmentation.

	**Manual**	**FreeSurfer 7.3**	**HIPS**	**ASHS**	**HippUnfold**	**HSF**
Segmentation time (min)	~300	678.58 (baseline)	N/A	30.10 ± 1.31	29.52 ± 2.35	1.64 ± 0.27

FreeSurfer segmentation time is computed as a reference point for a single HCP-aging subject (0.8 mm iso.). HIPS, ASHS, and HSF timings are mean ±std of segmentations on our complete test set of 25 subjects. Computations are conducted on a machine with an Intel(R) Xeon(R) Gold 6226R CPU @ 2.90 GHz, and an Nvidia A100 GPU.

**Figure 4:**
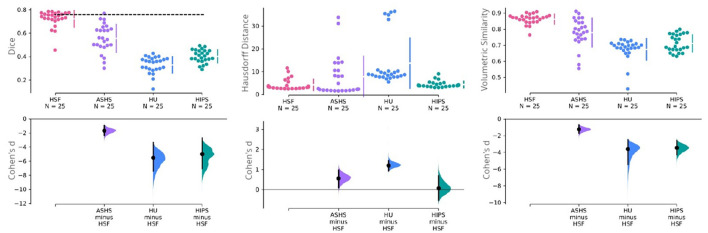
Cumming estimation plots comparing HSF (T2w) against ASHS (T1w and T2w), HIPS (T1w and T2w), and HippUnfold (HU) (T1w and T2w). The first row illustrates three performance metrics—the dice coefficient (higher is better), the Hausdorff distance (lower is better), and the volumetric similarity (higher is better). The vertical bars in this row represent the mean ±std for each metric group. The dashed line in this row represents the inter-rater reliability for manual segmentation obtained in the earlier study of Bouyeure et al. (2021). As this earlier study only computed the inter-rater comparison as the dice coefficient, it is not available for the other two metrics. The second row depicts the mean effect size (Cohen's d) with a black dot to facilitate statistical comparison between the groups. The black bars in this row represent 95% CIs for variability estimations. The 95% CIs are obtained through non-parametric bootstrap resampling to generate distributions of all possible effect sizes.

We used dice coefficient, Hausdorff distance, and volumetric similarity (Figure 4) with manual segmentations as benchmarking metrics. We found HSF to exhibit a significantly better DC than ASHS (*p* = 4*e*−6; hedge's *g* = 1.636), HIPS (*p* = 7*e*−9; hedge's *g* = 4.934), and HippUnfold (*p* = 7*e*−9; hedge's *g* = 5.440), with no differences between HippUnfold and HIPS.

Regarding HD, which is sensitive to outlier voxels in the segmentation, we found HSF performing on par with HIPS, but being better than HippUnfold (*p* = 7*e*−8; hedge's *g* = −1.184). Importantly, ASHS mainly penalized by poor segmentation results in a few observations although estimation statistics may suggest a difference between the two tools (Figure 4). Our statistical tests failed to reject the null hypothesis.

With respect to the VS, all three methods had similar volumes, but HSF was the closest to manual segmentations (VS = 0.862), better than ASHS (*p* = 2*e*−4; hedge's *g* = 1.210), HIPS (*p* = 9*e*−9; hedge's *g* = 3.391), and HippUnfold (*p* = 8*e*−9, hedge's *g* = 3.550). We found no differences between HIPS and HippUnfold.

After an extensive evaluation, we analyzed the disparities in segmentation quality compared to the T1w and T2w images on a subset of our test set where both contrasts were acquired using the same resolution, as outlined in Table 3. While the effect sizes were negligible, we found that T2w images tend to exhibit a slight inclination, with HSF producing segmentations closer to the manual ones, especially on the smallest regions, CA1, 2, and 3 (DC increased by 0.045, HD decreased by 2.386, and VS increased by 0.035).

**Table 3:** Comparison of the segmentations produced on T1w and T2w MRIs.

	**DC**			**HD**			**VS**		
**Label**	**T1w**	**T2w**	**Delta**	**T1w**	**T2w**	**Delta**	**T1w**	**T2w**	**Delta**
**DG**	0.850	0.900	**0.050**	4.880	2.540	**−2.340**	0.960	0.980	0.010
**CA1**	0.819	0.868	**0.049**	5.170	2.448	**−2.722**	0.907	0.952	**0.045**
**CA2**	0.781	0.828	**0.047**	4.400	1.952	**−2.448**	0.852	0.905	**0.053**
**CA3**	0.796	0.849	**0.053**	4.534	2.767	−1.767	0.868	0.924	**0.056**
**Sub**	0.830	0.859	0.029	5.492	2.787	–**2.705**	0.921	0.932	0.010

MRIs are coming from HCP-development, HCP- young adults, and HCP-aging. Those 15 subjects are a subset of our test set (**N** **=** 25). Those subjects are special cases where T1w and T2w MRIs are in the same space, with the same resolution. Deltas in bold denote significant differences at a p-value of < 0.05.

### 3.2. Human Connectome Project

#### 3.2.1. Lifespan development dynamics

**Figure 5:**
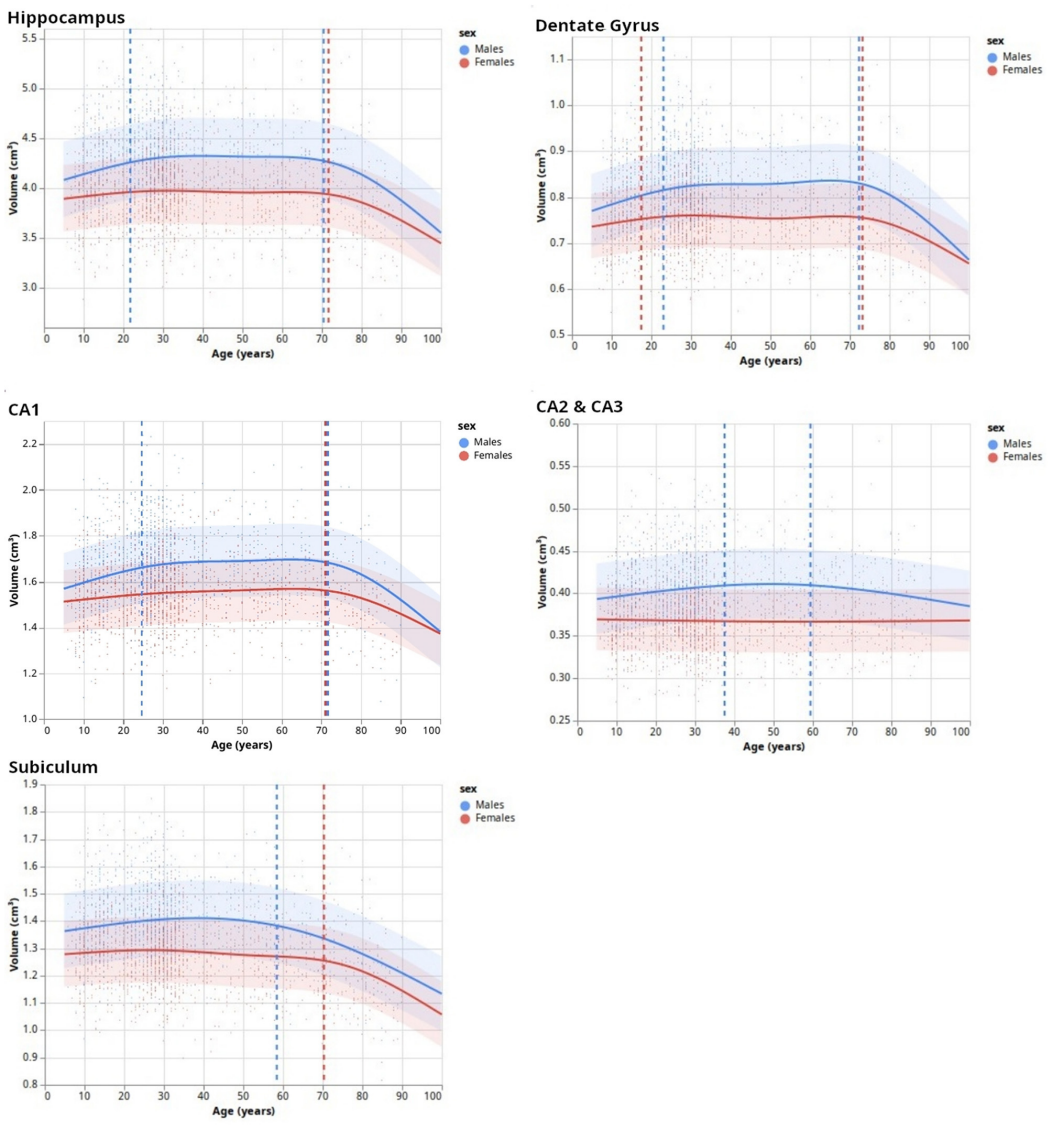
Lifespan dynamics of hippocampal subfields. Trend lines (surrounded by standard errors) are defined as natural cubic splines with a number of degrees of freedom minimizing an Akaike Information Criterion. Vertical dashed lines indicate inflection points.

After the HSF's retraining including new HCP subjects to ensure segmentation quality, we established lifespan trajectories (Figure 5) consisting of Natural Cubic Splines, from which we inferred inflection points reflecting lifespan critical periods. DG was the subfield whose developmental trajectory was the most correlated with age (*p* = 0.005). Total hippocampal volume was negatively correlated with age for both sexes starting from 70 years old (*p* = 0.03), which is also reflected in the subiculum (*p* = 2*e*−8). In addition to significant differences in volumes between sexes mostly during the “stable adulthood” period, except for CA2/3 (*p* = 0.120), we found differences between men and women during the “development” period in the DG (*p* = 0.01), and CA2/3 (*p* = 0.01), and during the aging period for the DG (*p* = 0.015) and CA1 (*p* = 0.04). Interestingly, we found differences in trajectories between men and women (i.e., interaction between age and sex), for the development period of CA2/3 (*p* = 0.017), for the aging period of the DG (*p* = 0.04), and before 60/70 years for the subiculum (*p* = 0.016).

#### 3.2.2. From head to tail: subfields' distribution

**Figure 6:**
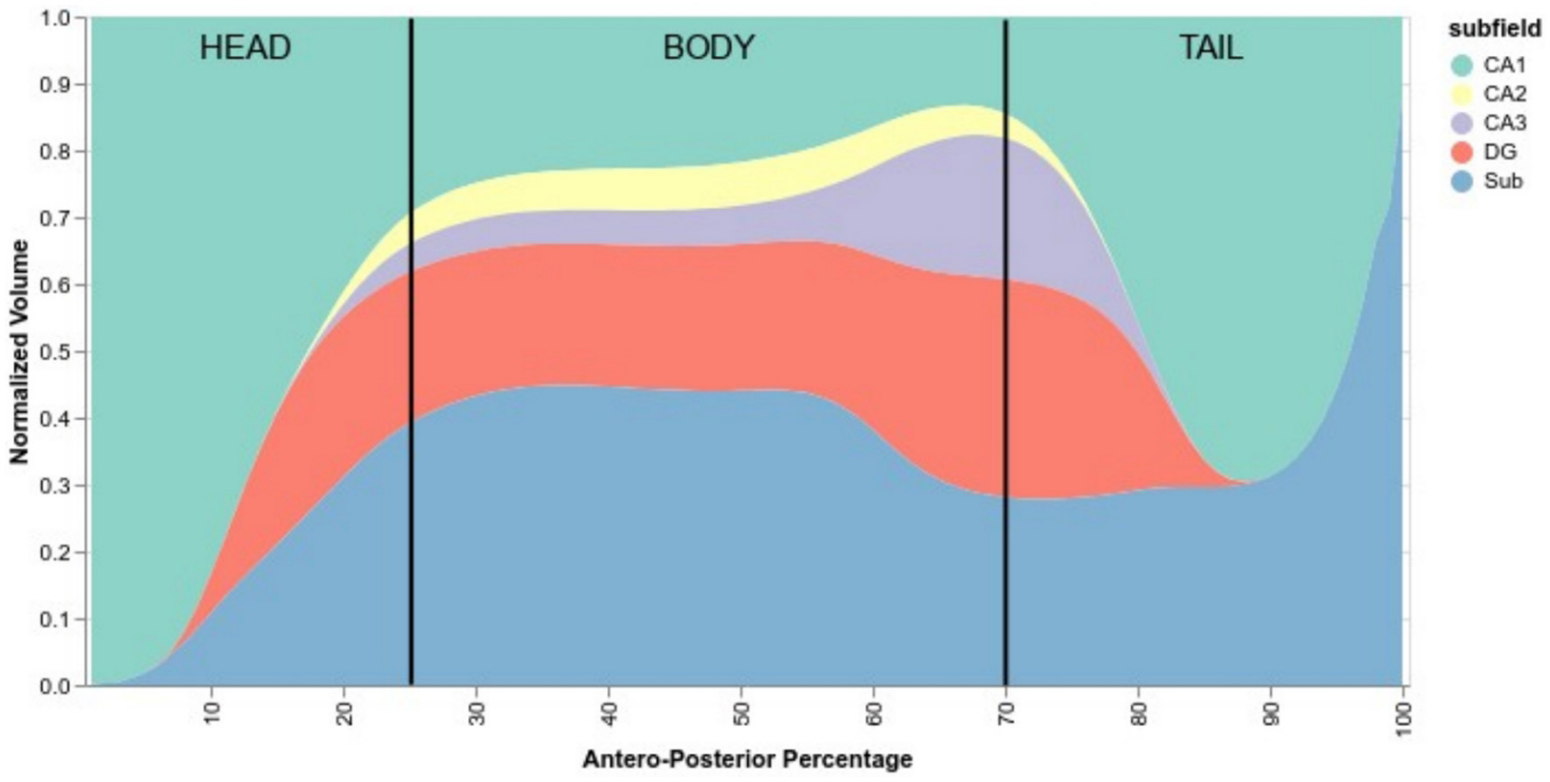
Normalized anteroposterior composition of subfields, going from 0% of the hippocampus (head) to 100% (tail). Vertical black lines are approximate delimiters of the head, body, and tail of the hippocampus.

Delineating the subfields in the head and the tail of the hippocampus is a complex task, with some protocols not even delineating subfields in the tail. Due to the peculiar training methods, we trained HSF to segment the head and the tail even when there was no ground truth subfield segmentation in these regions. Using HSF, we created an overall normalized anteroposterior distribution of subfields across all three HCP datasets (Figure 6). We found no anatomical differences between lifespan periods and sexes.

According to HSF, the hippocampal head starts mostly with CA1, quickly followed by the subiculum and then the DG before the hippocampal body. After the body, CA2 and CA3 start to disappear and then followed by the DG. The tail comprises mostly subiculum, CA1, and a small portion of DG which disappears near the middle of the tail.

## 4. Discussion

This study had two main goals: 1/to introduce a new segmentation tool for the hippocampal subfield based on machine learning named hippocampal segmentation factor (HSF), which leverages the latest advances in computer vision, and 2/to study lifespan volumetric trajectories of hippocampal subfields in healthy individuals using the proposed tool. We developed and validated HSF, and demonstrated that it is faster than all previous tools while offering a better segmentation quality closer to manual segmentation. Then, applying our tool to data from 3,750 individuals (HCP-development, HCP-young adults, and HCP-aging), we show that hippocampal subfields have different volumetric trajectories across the lifespan. These trajectories are non-linear, and inflection points differ between males and females in accordance with prior literature (16).

First of all, we validated HSF in comparison to ASHS, HIPS, and HippUnfold. When looking at the DC, it has to be noted that, even in the absence of histological ground truth, HSF matches the inter-rater agreement (Figure 4). Moreover, its scalability benefits out-of-the-box from the latest advances in computing due to the open neural network exchange (ONNX) ecosystem and NeuralMagic's DeepSparse inference engine. HSF shows an unprecedented segmentation speed which makes it particularly suited to the processing of big datasets such as the HCP. The bootstrap aggregation strategy, coupled with the test-time augmentation, makes HSF more robust than ASHS and HippUnfold as suggested by our results, with a lower variance with respect to the DC, HD, and VS (Figure 4). One feature of interest is the ability of HSF to segment both T1w and T2w images. Our investigation yielded superior quality segmentations through the utilization of T2w images—a result that aligns with the existing literature. It is important to note, however, that our dataset contained a larger quantity of T2w images compared to T1w images. Therefore, we are unable to definitively conclude whether the observed disparities in quality are a direct result of superior T2w contrast or a potential bias within our dataset. However, because each tool was trained using data segmented with different protocols, it is difficult to compare their accuracy, especially regarding the boundary between CA1 and the Subiculum (Yushkevich et al., 2015a). As HSF learned from multiple datasets, we interpret its segmentation as following a consensus between multiple segmentation guidelines, even if our results show it is very close to Barron's protocol (Berron et al., 2017). All tools segment the head and the body of the hippocampus in a similar manner, except HIPS which after manual verification, did not seem to respect the hippocampal subfields' boundaries visible to the naked eye. HippUnfold underperforms compared to HSF and ASHS because it overrepresents CA2 and CA3 in the tail. The way HSF learned to segment the hippocampal tail (Figure 6) is very similar to the histology-based tail segmentation proposed by Dalton et al. (2017), Flores et al. (2020), which both differ from Barron's protocol. There is no histological ground truth to support the superiority of HSF over HippUnfold regarding tail segmentation. If HSF was to be proved wrong regarding this particular point, future investigators could easily add new deep learning models to HSF's Model Hub in a plug-and-play fashion. Ever since the most recent launch of FreeSurfer 7, the original authors (Iglesias et al., 2015) have been endeavoring to enhance their segmentation pipeline of the hippocampal subfields. Due to the fact that this updated version is still untested and limited, it has not been integrated into our benchmark because of the current limitation to low-resolution T1 images. Thus, we highly suggest that future studies thoroughly examine this novel update as soon as it exits the beta stage.

After validating HSF, we segmented and analyzed hippocampal ROIs obtained from the HCP-development, HCP-young adults, and HCP-aging datasets. This allowed us to study the developmental trajectories of hippocampal subfields during the lifespan with a bigger age range than previous studies [e.g., (Yang et al., 2013; Bookheimer et al., 2019)]. Our model selection of NCS based on AIC found three main patterns. The first pattern, as expected, divided the hippocampus developmental trajectory into three main periods: growth, stabilization, and decay (GSD). This is the overall developmental pattern of the hippocampus, showing a maximal volume at approximately 20 to 25 years old, which is lower than some previous studies [e.g., (Yang et al., 2013)] but this may be due to the finer resolution of our model, thus allowing the observation of three distinct trends. After the stable period, we found a significantly negative correlation between hippocampal volume and age from 70 years old onwards, which is approximately 8 years later than previously found (Ziegler et al., 2012; Yang et al., 2013; de Flores et al., 2015). As previously, this may be caused by modeling artifacts, survivor bias, or inclusion bias in the used datasets (inclusion of “super-healthy” individuals with better aging than the general population). This GSD trajectory was observed in DG and CA1, which is consistent with previous studies showing growth during infancy and childhood (Lavenex and Banta Lavenex, 2013; Lee et al., 2014; Ellis et al., 2021), [up to a 2-fold increase in size for DG (Bachevalier, 2013)]. Moreover, the inflection points of DG and CA1 were very similar to those of the total hippocampus (Figure 5). However, we observed different trajectories for CA2/3 and the subiculum. Although the literature suggested a volumetric increase of CA2/3 (Lavenex and Banta Lavenex, 2013; Lee et al., 2014), we found this structure to be the most stable across the lifespan with no clear trend. This may be due to an insufficient resolution, forcing us to merge CA2 and CA3, thus averaging their dynamics. Another possible factor might be a too-noisy segmentation because of partial volumes resulting in a lack of sensibility to detect fine changes in these small and complex regions. Finally, our results for the subiculum are consistent with the literature: mostly flat (i.e., absence of correlation of volume with age) or a slight quasi-linear negative correlation between age and volume (Ziegler et al., 2012; Lee et al., 2014; de Flores et al., 2015; Foster et al., 2019). Our bigger age range and finer model allow us to refine those characteristics: by examining our results, we found a plateau, no correlation between age and volume, until the age of 60~70 years after which a fast decay happens similar to other subfields. Overall, this suggests that the DG, followed by CA1, is the most affected by development and aging. Most of the development of the subiculum appears to happen before the age of 5, which would relate to mnesic developments (Bouyeure and Noulhiane, 2021). While the subicular volume is positively correlated with the learnings of the when, where, and what components of episodic memory (Chi et al., 2022), prior studies found correlations between episodic memory and subiculum only up to 5 years old, which might be caused by the earlier maturation of the monosynaptic pathway (Canada, 2020). If the subiculum appears to mature earlier, it also decays earlier than others, which suggests that it might be a relevant biomarker for the early identification of age-related cognitive impairments. Furthermore, given that our findings are largely consistent with prior research, this serves to strengthen the validity of HSF, our novel segmentation tool.

Finally, besides sexual dimorphism with men having, over the stable part of their life, bigger hippocampal subfields than women, we found differences in developmental trajectories of hippocampal subfields between men and women. These are debated in the literature since some studies did not find interactions between volume, sex, and age (Sullivan et al., 2005; Mueller et al., 2007), while others did (). The present study suggests a complex relationship since we did not find such an interaction for all subfields. We found significant differences only for the growing period of the DG and CA2/3 with a faster growth in men than in women. This may be due to gonadal hormones modulating neoneurogenesis and increasing adult-born cells' survival in the DG (Galea et al., 2006; Spritzer and Galea, 2007; Hamson et al., 2013). However, this literature suggests that this interaction also exists in CA1 (Leranth, 2004; Islam et al., 2020), which was not the case in our study. Interestingly, we also observed a stronger negative correlation between age and volume for the DG and CA1 in men than in women. Overall, our results add to the literature and reconcile previous results on the lifespan volumetric trajectories of hippocampal subfields.

Our study suffers from several limitations. First, the lack of a standardized protocol to segment the hippocampal subfields negatively affects the way algorithms will learn to segment. This is partly solved by learning from a consensus between guidelines, but we lack a better *in vivo* ground truth than the one provided by manual segmentations. Then, volume might not reflect all the age-related changes in hippocampal structures. Although we found no anteroposterior differences between subjects, we believe it is critical to go beyond volumetric analysis and assess additional information, such as shape as suggested by Yang et al. (2013), Voineskos et al. (2015), and Lynch et al. (2019) or other complementary measures gathered through diffusion imaging, or even quantitative T1 relaxation maps, a proxy for intracortical myelin (Vos de Wael et al., 2018).

Therefore, while the hippocampal subfields are critical in the physiology of episodic memory, the lack of efficient segmentation tools hinders the use of large datasets to study their role in health and disease. Here, we introduced a new segmentation tool, HSF, robust to changes in populations, and acquisition parameters such as contrast, resolution, or magnetic field intensity. After its validation against other existing tools (ASHS, HIPS, and HippUnfold), we used it to segment large datasets (HCP-development, HCP-young adults, and HCP-aging) in order to model volumetric trajectories of the hippocampal subfields from 5 to 100 years old. Our volumetric analysis has shown that most subfields except the subiculum are positively correlated with age until the early 20s, and that the most correlated subfield is the dentate gyrus. This study also found a major inflection point at approximately 70 years old (even earlier in the subiculum) where a fast and significant volumetric decrease occurs. Our study has yet to be correlated with evaluations of mnesic performances, which could help to validate subicular volumes as a relevant biomarker for the early diagnosis of age-related cognitive decline.

## Data availability statement

The datasets with the exception of HCP datasets presented in this article are not readily available because participants provided consent only for using their data under the supervision of the principal investigator. Requests to access the datasets should be directed to MN.

## Ethics statement

The studies involving human participants were reviewed and approved by CPP 2011-A00058-33. Written informed consent to participate in this study was provided by the participants' legal guardian/next of kin.

## Author contributions

CP: conceptualization, methodology, software, formal analysis, data curation, writing—original draft, and visualization. AB: investigation, resources, data curation, writing—review and editing, and funding acquisition. SP and MF: data curation and writing—review and editing. AG: methodology. ED: methodology and writing—review and editing. MB: resources, data curation, and writing—review and editing. FL: writing—review and editing. MN: conceptualization, investigation, resources, writing—original draft, supervision, project administration, and funding acquisition. All authors contributed to the article and approved the submitted version.
